# Complete corporeal preservation clitoroplasty: new insights into feminizing genitoplasty

**DOI:** 10.1590/S1677-5538.IBJU.2020.0839

**Published:** 2021-01-10

**Authors:** Nicolas Fernandez, Julián Chavarriaga, Jaime Pérez

**Affiliations:** 1 Pontificia Universidad Javeriana Hospital Universitario San Ignacio Division of Urology Bogota Colombia Division of Urology, Hospital Universitario San Ignacio, Pontificia Universidad Javeriana, Bogota, Colombia;; 2 Fundacion Santa Fe de Bogota Department of Urology Bogota Colombia Department of Urology, Fundacion Santa Fe de Bogota, Bogota, Colombia;; 3 University of Washington Seattle Children's Hospital Division of Urology SeattleWA United States Division of Urology, Seattle Children's Hospital, University of Washington, Seattle, WA, United States

**Keywords:** Surgical Procedures, Operative, Adrenal Hyperplasia, Congenital, Clitoris

## Abstract

**Introduction:**

46,XX Congenital adrenal hyperplasia (CAH) remains the first cause of genital virilization and current surgical techniques aim to restore female aspect of genitalia while preserving dorsal neurovascular bundle but not at the expense of not preserving erectile tissue. We aim to report our experience with a new surgical technique for clitoroplasty, completely preserving corporeal bodies, neurovascular bundles without dismembering the clitoris, in four patients with over a year follow up.

**Materials and Methods:**

After IRB approval four patients with 46,XX CAH and Prader 5 and 3 external genitalia, underwent feminizing genitoplasty. Complete preservation of erectile tissue was accomplished without a need to dissect dorsal neurovascular bundle. Glans size allowed no need for glanular reduction and there was no need to dismember the corporeal bodies.

**Results:**

Four patients 12 to 24-months-old underwent complete corporeal preservation clitoroplasty (CCPC), mean age was 18.5 months, mean follow up was 10.25 months. Vaginoplasty was performed in all patients with partial urogenital mobilization (PUM) and Urogenital Sinus flap (UF), only one severely virilized patient required a parasagittal pre-rectal approach to mobilize the vagina. We had no complications until last follow up.

**Conclusion:**

To our knowledge, we are introducing the concept of CCPC without the need of disassembling the corporeal bodies, neurovascular bundle and glans. It stands as a new alternative for feminizing genitoplasty with complete preservation of erectile tissue and no dissection of neurovascular bundle. Although there is still lacking long-term follow-up, it represents a new step in conservative reconfiguration of the external virilized female genitalia.

## INTRODUCTION

One of the most complex problems of pediatric urology is the surgical management of disorders of sex development (DSD). Congenital adrenal hyperplasia (CAH) is the most common 46,XX DSD with a prevalence of 1 in 15.000 newborns (1). The most frequent cause is enzymatic deficiency of 21-hydroxilase, followed by 11β-hydroxylase. The lack of these enzymes manifests with varying degrees of virilization of the external genitalia as the result of excess androgenic metabolites (1–3).

Management of DSDs should be conducted by a transdisciplinary team that allows proper genetic counseling, accurate diagnosis, classification of the DSD, individualized surgical planning and a comprehensive decision-making process involving the family (1, 4). Feminizing genitoplasty (FG) still remains a topic of controversy in many countries and cultures. Most families opt for early surgery, considering the positive implications for children's psychosocial development, relieving parents distress and restoring “normal” external genital configuration. Szymanski et al. evaluated parental decisional regret after FG, they reported that 20.5% of parents, reported some regret, in contrast no parent would have chosen again delayed surgery (1, 4–6).

FG generally involves: labioplasty, vaginoplasty, urethroplasty and clitoroplasty, the latter has been the main focus of discussion for pediatric urologists and reconstructive surgeons given the crucial role that clitoris has in female sexuality. Most authors agree that every effort should be make in order to preserve clitoral innervation and function. Clitoral amputation techniques have been abandoned, and burying the corpora underneath the pubis remains uncertain considering the potential for painful clitoral erections. At present most surgeons preserve the neurovascular bundle (NVB) and erectile tissue is excised (7–9). More recently, Pippi Salle described the corporeal sparing dismembered clitoroplasty (CSDC) technique in which the NVB is separated from the glans and corporeal bodies, which then are buried in the neo-labia majora without the erectile tissue (2). To our knowledge there are no previous descriptions of a complete corporeal preservation clitoroplasty (CCPC) technique and hereby we present our surgical approach.

## MATERIALS AND METHODS

After institutional ethics board approval, IRB number 20190531-805, following extended discussion with the parents, health care providers and after a transdisciplinary meeting for disorders of sex development (DSD) records of all patients that underwent FG between 2018-2019 were reviewed, we identified 4 patients who underwent CCPC. All patients had never undergone surgery prior to this intervention. Patient clinical and sociodemographic characteristics are listed in Table-1.

**Table 1:** Clinical Characteristics of 4 patients with Congenital Adrenal Hyperplasia.

Patient No.	Age (Month)	Diagnosis	Clitoral lenght	UGS Size (Cm)	Urethra	Vagina	Prader	Clitoroplasty	Vaginoplasty	Follow-Up (Month)
1	24	CAH	4.5	5.5	0.7	2.5	5	CCPC	PUM+USF	19
2	12	CAH	2	1	2	3	3	CCPC	PUM+USF	16
3	14	CAH	5	1	2	3.5	3	CCPC	PUM+USF	11
4	24	CAH	5.5	1	2.5	4	3	CCPC	PUM+USF	11

**UGS:** Urogenital sinus,

**CAH**: Congenital adrenal hiperplasia,

**CCPC:** Complete corporeal preservation clitoroplasty,

**PUM:** Partial urogenital mobilization,

**USF:** Urogenital Sinus flap

### Surgical Technique

**Figure 1:**
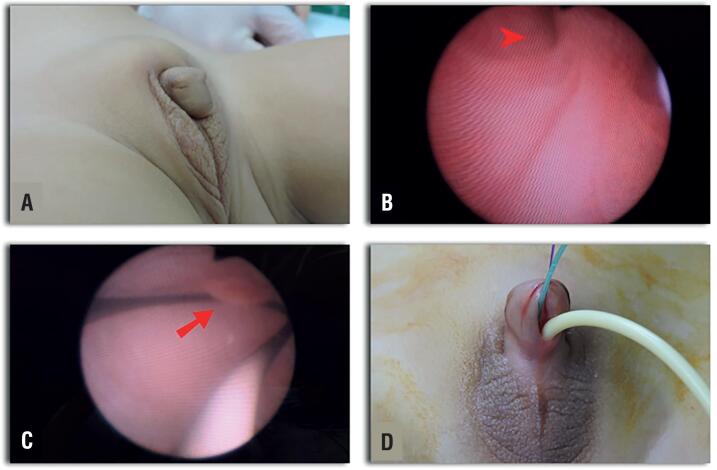
Patient 1, 24 months old. A) Virilized External Genitalia, Prader V. B) Well-defined (male-like) external sphincter with a verumontanum appearance distal to the vaginal confluence (Arrow head) C) Vagina and Cervix (Arrow) D) A 3 fr Fogarty catheter in the bladder and a 6 fr Foley catheter in the vagina both going through the urogenital sinus (UGS).

Patients were prepped and draped in the usual sterile fashion, allowing the possibility of changing from prone to supine without a need to re-drape the patient (10, 11). The procedure was then started with an endoscopic evaluation of the urinary tract including vaginoscopy. The urogenital sinus (UGS), vaginal and urethral length were measured (Figure-1).

**Figure 2:**
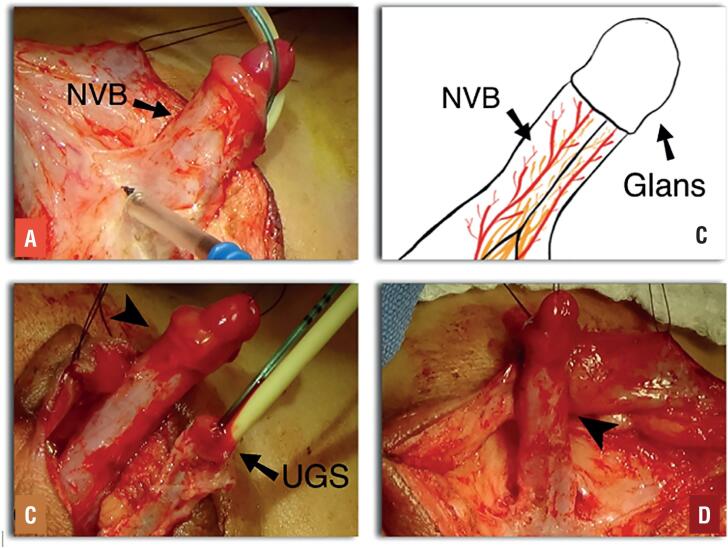
Patient 1, 24 months old A) and B) Clitoral dissection preserving the dorsal neurovascular bundle (NVB) intact C. The clitoris and the NVB (Arrow head) dissected from the UGS (Arrow). D) Non-dismembered clitoris (Arrow head) with its NVB preserved.

**Figure 3:**
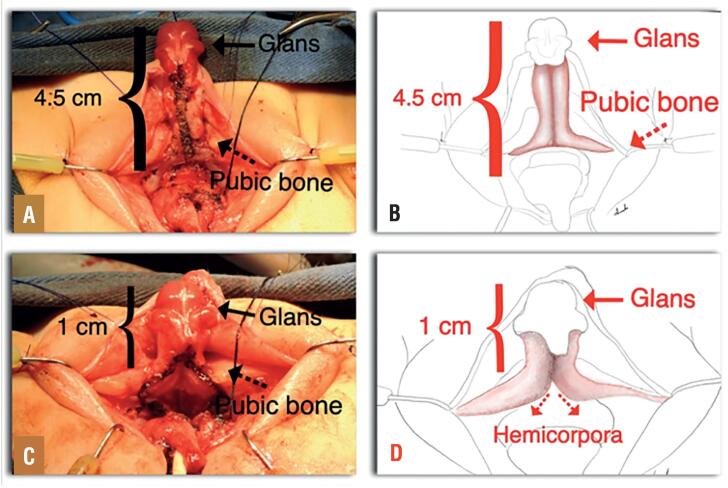
Patient 2, 12 months old. A) and B) Detachment of the two hemicorpora in the midline (Arrow), up to the middle third of the phallic shaft. C) and D) Completed, corporeal sparing clitoroplasty showing a 3.5cm reduction in clitoral length. The proximal two thirds of each hemicorpora were laterally mobilized and anchored to the pubic bone (Red Dotted arrows).

A 3 Fr Fogarty catheter was left inside the bladder and a 6 Fr Foley inside the vagina. In a severely virilized patient and considering the high confluence and long common channel, a parasagittal pre-rectal approach was performed in prone position. Extensive dissection was then possible. The other 3 patients underwent an anterior approach dissecting from the common channel of the UGS to the vagina. Anterior pubo-urethral ligaments were preserved in all cases. Once the common channel was dissected, an incision was made at the junction between the common channel and the vagina. Multiple repair stitches were placed to secure traction without tearing vaginal tissue. The clitoris was then degloved and the NVB was never disrupted and left intact. On the ventral aspect, the common channel was dissected off the corporeal bodies (Figure-2). Once a complete mobilization of the common channel was achieved, we brought our attention to the corporeal bodies, which were dissected and divided in the midline on a distal direction starting at the bifurcation (Figure-3).

The glans, dorsal NVB and perforating branches entering at the dorsal junction between the glans and corpora were not disturbed in order to preserve complete sensitivity and vascularity to the clitoral glans. The splitted proximal two thirds of each hemicorpora were laterally mobilized and anchored to the pubic bone with 6-0 polypropylene avoiding overlapping or folding the distal aspect of the corporeal bodies. The aim was to have a protruding 1cm clitoris after this maneuver (Figure-3).

## RESULTS

Four patients 12 to 24-months-old underwent CCPC as described earlier. Mean age was 18.5 months, all patients had molecular confirmed diagnosis of CAH, three by deficit of 21œ-hydroxylase and one with 11β-hydroxylase deficit. Median clitoral length was 4.75cm (IQR 2-5.5). Mean follow-up was 10.2 months. Vaginoplasty was performed in all patients with partial urogenital mobilization (PUM) and Urogenital Sinus flap (UF) technique. Three patients had Prader 3 external genitalia and one severely virilized girl was Prader 5.

**Figure 4:**
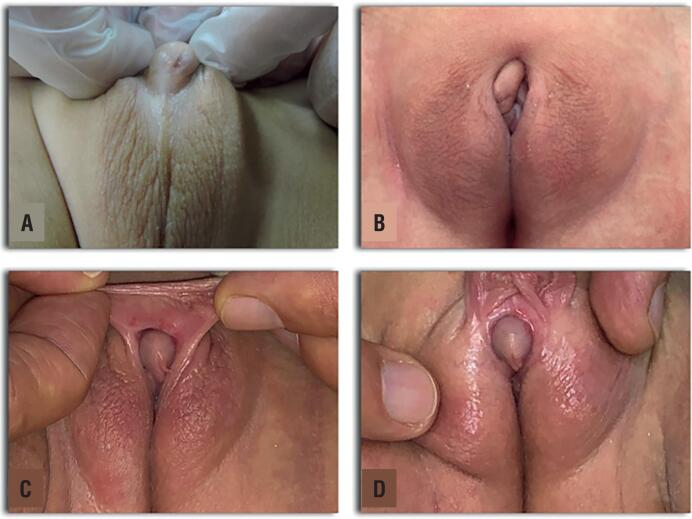
A) Patient 3, 14 months old 46,XX with virilized external genitalia showing a Prader III configuration. B) External genitalia appearance at ten months follow-up after complete corporeal preservation clitoroplasty. C) and D) Patient 1, 24 months old, External genitalia appearance at 18 months follow-up after clitoroplasty.

The postoperative course was uneventful in all patients, all of them were discharged without complications on the second postoperative day. Epidural catheter was placed at the beginning of the cases and kept in place until the first postoperative day. Foley catheter was removed on the seventh postoperative day. Based on parent's perspective and the surgical team opinion, a satisfactory phenotypic appearance was achieved at the follow-up visits. Postoperative results are shown on Figure-4. All patients continued to be followed up until puberty and were instructed to adhere to endocrinological treatment.

## DISCUSSION

Despise all efforts to understand DSD, genital ambiguity and its surgical management remains challenging. In the last consensus statement on the management of DSD, it was suggested to perform early surgery and UGS repair in girls with severe virilization (9). On the opposite side of the spectrum, a number of advocacy groups, question the benefit of early surgery and the ethical issues involving parents and physicians rights making decisions for the children. The recommendation is that the decision has to be made by a transdisciplinary team with transparency, presenting all pros and cons of surgery versus observation without withholding any information from families (1, 4–6, 12).

Clitoroplasty has been part of the surgical management for virilized external genitalia for over a century (3, 9, 11, 13). At present, most DSD surgeons believe that it should not be addressed as a single procedure but as part of a complete reconstruction of the external genitalia and the UGS. Vaginoplasty regardless of vaginal configuration is best combined with labioplasty and clitoroplasty in a single-stage procedure. The 4th World Congress of the International Society of Hypospadias and Disorders of Sex Development Surgery and the American Academy of Pediatrics, suggested performing the surgery before 2 years of age and offering a single-stage, rather than multi-stage interventions (8, 9, 14).

Clitoroplasty has undergone a significant evolution since H.H. Young first described it in 1934. Initial attempts to not amputate the clitoris were called reduction techniques and they implied reducing corporal length without preserving the glans (16). Later the clitoridectomy was conceived by Hampson and Money supported by the theory that the clitoris had no function (13, 15–18). It was just a matter of time until the importance of the clitoral function regarding female sexuality was discovered and all the attempts to preserve the clitoris started to become a standard practice (18–20). Thereafter, Lattimer introduced his technique, where he suggested preserving the tip of the glans, reducing the erectile tissue and relocating the clitoris underneath the pubis (21). Randolph and Hung modified Lattimer's technique and proposed resection of the exposed shaft of the corpora cavernosa preserving the glans (22). It was not until Kogan reported his subtunical reduction technique that preventing damage to the NVB became the cornerstone of the clitoroplasty (23). Roughly there are three techniques for clitoroplasty: clitoridectomy, recession clitoroplasty and reduction clitoroplasty, in none of these techniques preservation of the erectile tissue of the cavernous bodies was intended (11, 13).

Pippi Salle described in 2007 his CSDC, the aim of this revolutionary technique was to dissect the NVB and the glans off the corpora cavernosa, splitting the corpora in the midline leaving two hemicorpora and burying them in a dartos pouch in the labia majora, after removing all erectile tissue. This technique preserves the corpora cavernosa considering that up to 5% of the patients with CAH may have gender dysphoria later in life and a neophallus could be created from the buried corpora cavernosa (24). To our knowledge, at present, there had been no cases reported in literature where feminizing genitoplasty for CAH has to be reverted (2, 25).

To our knowledge, our technique has never been described before and represents the first of its kind. As we describe it, we intend to spare the corpora cavernosa erectile tissue of the clitoris without disassembling the clitoris and leaving the dorsal NVB untouched. Clitoral erections, sensitivity and vascularity to the clitoris could be harmed by dismembering the clitoris. One of the limitations of burying the clitoris is the possibility of clitoral pain with erections as has been described in reduction clitoroplasty (2, 3, 11, 13). In the CCPC we do not remove erectile tissue of the cavernous bodies and we partially plicate the hemicorpora in order to reduce clitoral length, leaving erectile tissue and NVB intact, leading us to believe clitoral erections are possible and should not be painful. Unfortunately, long-term follow-up until puberty has not been completed at the time this article was written and we will not know the outcome of the surgery until these patients are old enough to be sexually active, which could represent a limitation of our article; another limitation is the small sample and short follow-up period to date.

## CONCLUSIONS

The complete corporeal preservation clitoroplasty (CCPC) stands as a new alternative in the feminizing genitoplasty armamentarium, although there is still lacking long-term follow-up on clitoral function and phenotypic appearance after puberty, it represents a new step in conservative reconfiguration of the external virilized female genitalia. The irreversibility of the dismembered clitoroplasty no longer remains a concern for the patients and their families, if wanted, the surgery could be reverted at any time.
